# The use of autologous fascia lata graft in the laparoscopic reinforcement of large hiatal defect: initial observations of the surgical technique

**DOI:** 10.1186/s12893-015-0008-2

**Published:** 2015-03-11

**Authors:** Milos Bjelovic, Tamara Babic, Bratislav Spica, Dragan Gunjic, Milan Veselinovic, Violeta Bascarevic

**Affiliations:** University of Belgrade, School of Medicine, Belgrade, Serbia; Department of Minimally Invasive Upper Digestive Surgery, Clinic for Digestive Surgery, Clinical Center of Serbia, Belgrade, Serbia; Department of Plastic Surgery, Special Hospital Banjica, Belgrade, Serbia

**Keywords:** Fascia lata, Hiatal hernia, Recurrence, Reinforcement, Tissue transfer

## Abstract

**Background:**

Even though there is no consensus, many authors believe that in the cases of large hiatal defects, structurally altered crura and/or absence of peritoneal lining, a crural reinforcement should be performed. Reinforcement could be performed with different techniques and different type of mesh, either synthetic or biologic. The disadvantages of mesh repair include the possibility of serious complications and increased costs especially in the usage of composite or biologic mesh.

**Methods:**

The study includes 10 cases of reinforced primary suture line of the pillars with autologous fascia lata, in elective laparoscopic repair of the giant PEH with a large hiatal defect and friable crura. After intraopreative confirmation of the large hiatal defect (hiatal surface area of more than 8 cm^2^) and friable crura, an autologous fascia lata graft was harvested in the usual manner and placed in on-lay fashion to reinforce the pillar suture line. We analyzed surgical technique, complications, and initial follow-up of the patients.

**Results:**

Average hiatal surface area (HSA) in our series was 10.6 cm^2^ (range 8.1 to 14.4 cm^2^). The average duration of operation was 203.9 min/3.4 hours (range 160–250 min). Except for a mild hematoma in the harvesting region that resolved spontaneously, there were no procedure related complications and 30 days mortality rate was zero. The average postoperative length of stay was 6.5 days (5–8 days). Out of 10 patients, 5 completed the annual follow-up visit, while 8 completed a 6- month follow-up visit. So far there is no hernia recurrence and/or problems with swallowing function. However, one patient has felt a mild discomfort in the harvested region that does not influence normal daily activities.

**Conclusions:**

Autologous fascia lata graft hiatal reinforcement represents a technically feasible, easy, and available option for the on-lay reinforcement of large hiatal defects with friable crura in the laparoscopic repair of giant PEHs.

## Background

Several studies have addressed the issue that after retroesophageal cruroraphy in the case of a giant paraesophageal hernia (PEH) recurrence rates could be expected in up to 43% of cases [1,2]. There are two main technical causes of recurrent hiatal hernia: an unrecognized secondary shortened esophagus and insufficient hiatal closure. Even though there is no consensus, many authors believe that in the cases of large hiatal defects, structurally altered crura and/or absence of peritoneal lining, a crural reinforcement should be performed [3,4]. Several technical options were proposed; amongst them the use of mesh reinforced hiatoplasty. However, there is still a pending question regarding mesh-related complications [5]. Another issue is mesh cost, as well as the issue of using heterologous tissue transfer in the case of biologic mesh usage [6].

In 2012, a group from Pecs, Hungary, offered a biologic alternative by performing fascia lata graft hiatoplasty on animal model [7]. In 1931 Janes published the use of autologous fascia lata graft in the treatment of post-traumatic diaphragmatic hernia [8]. In 1968 Brain published the use of autologous fascia lata graft to create a new phrenoesophageal ligament in the transthoracic repair of hiatal hernia [9]. At our Department the first use of autologous fascia lata graft to reinforce the primary suture of the pillars in the case of a large hiatal defect with friable crura was performed in April 2013. In this series we present a biologic hiatal reinforcement with an autologous fascia lata graft as a technically feasible, easy, and available option for reinforcing large hiatal defects with friable crura in the laparoscopic repair of giant PEH.

## Methods

Laparoscopic repair of hiatal hernias has been routinely performed since 2004 at the Clinic for Digestive Surgery, Clinical Center of Serbia. Recently we performed in an average of 35 laparoscopic cases of hiatal hernia a year, with a giant PEH as the most prevalent hernia type. At our Department, innovative biologic hiatal reinforcement with autologous fascia lata graft in cases of large hiatal defect with friable crura has been the standard procedure since April 2013.

The study included 10 patients with giant PEH and a large hiatal defect with friable crura, which underwent elective laparoscopic hiatal hernia repair at the Department for Minimally Invasive Upper Digestive surgery, Clinic for Digestive Surgery of the Clinical Center of Serbia in Belgrade from April 2013 to November 2014.

Written informed consent was obtained from all patients prior to surgical intervention. All patients underwent a standard surgical technique of giant PEH repair [10]. After intraoperative confirmation of the large hiatal defect (hiatal surface area of more than 8 cm^2^) and friable crura, autologous fascia lata graft was harvested in a usual manner and placed in on-lay fashion to reinforce the pillar suture line.

Control barium radiography was routinely performed on the first postoperative day followed by clear liquid diet, with exception of patients who underwent the esophageal lengthening procedure when barium radiography was performed on the postoperative day four. After hospital discharge, the first check-up was performed a month after surgery. Then, six months after surgery symptom evaluation and contrast radiography with barium meal were performed. The standard post-operative annual check-up included symptoms evaluation, contrast radiography with barium meal and upper flexible endoscopy.

### Surgical technique

The patient was placed in a supine position with trocar position adopted from J.D. Luketich [11]. Procedures started with a complete reduction of hernia and sac excision. The next step was extensive mediastinal esophageal dissection with vagal preservation. The gastroesophageal junction fat pad was dissected, with intraoperative evaluation of esophageal length in a tension free manner, and wedge gastroplasty in the case of the secondary shortened esophagus [10,12]. The antireflux procedure included a floppy Nissen fundoplication using 2–0 non-absorbable interrupted sutures. The size of the hiatal defect was assessed by the intraoperative measurement of hiatal surface area (HSA), as suggested by Granderath et al. [13]. HSA of more than 8 cm^2^ with the friable crura was the indication for fascia lata reinforcement.

**Figure 1:**
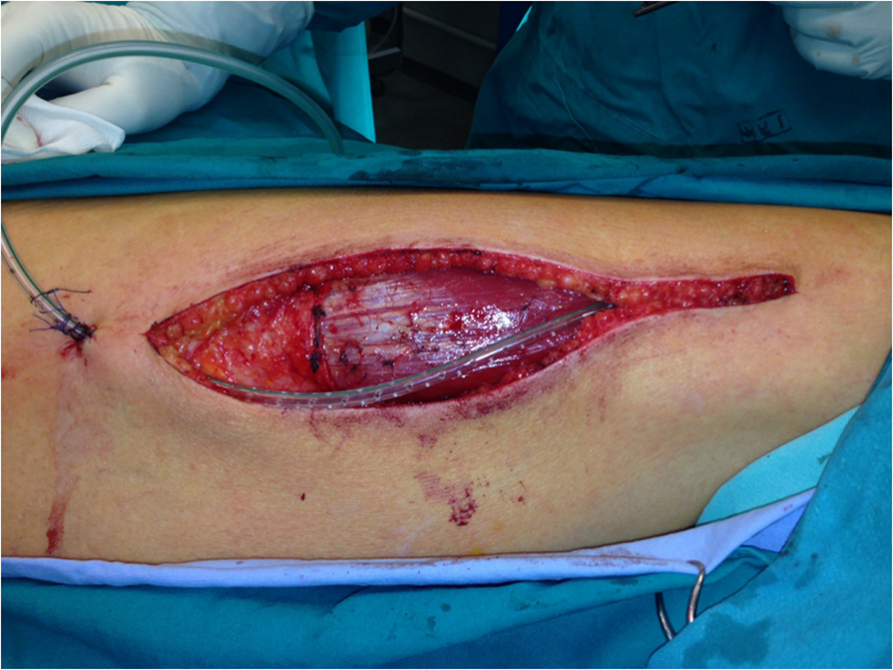
**Harvesting region.**

Autologous fascia lata graft was harvested from the right thigh of the patient. The incision was placed at the line that connects the lateral tibial epicondyle and the femoral greater trochanter approximately 5 cm above lateral tibial epicondyle (Figure 1). Afterwards, a careful dissection was performed with special attention paid to avoid the injury of the iliotibial tract. The harvested autologous fascia lata graft measured approximately 10×8 cm in diameter. The meticulous hemostasis was performed in which the coagulation of perforator veins was mandatory. The subcutaneous drainage was optional, based on the assessment of the operating surgeon and afterwards the wound reconstruction was performed. The autologous fascia lata graft was placed in sterile saline solution.

**Figure 2:**
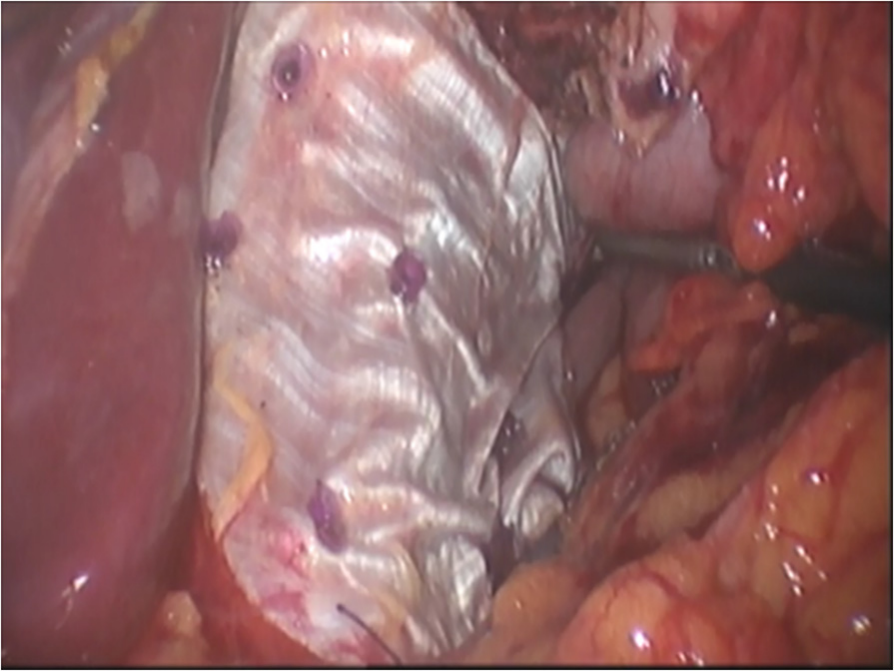
**Autologous fascia lata graft reinforcement of the large hiatal defect; fascia lata in place.**

Primary retroesophageal suture of the pillars was performed. For severely disrupted hiatus a complex reconstruction is required. In such a case the left crus is plicated to normalize the crural length, permitting a standard hiatal reconstruction [14]. Afterwards, an autologous fascia lata graft was placed in on-lay fashion and fixed with biodegradable tacks (Figure 2). There is an issue regarding fascia lata fixation. Solid and slippery surface of the graft, compared to mesh structure, may cause difficulties. However, a proper dissection of the retro-esophageal space allows enough room for the fascia lata deployment. After a proper positioning of the graft, the initial fixation is crucial. First tacks are placed on the most proximal part of the graft allowing the fascia to fall as a curtain over the crural region. While proximal tacks keep the fascia in place, in a step-by-step manner, additional tacks with the fascia lata underneath should be pressed against the surface. At that point, using the grasping forceps, the graft is lifted and folded to check the place of the fixation.

## Results

The outcomes of 10 cases of the laparoscopic repair of the giant PEH along with autologous fascia lata graft hiatoplasty from April 2013 to November 2014 were included. Demograpic details, surgical procedure and postoperative course related details are listed in Table 1.

**Table 1:** **Demographic, surgical procedure and postoperative course details**

**Features**	**No (%)**	**Average (range)**
**Gender**		
Males	2 (20.0%)	
Females	8 (80.0%)	
**Age**		64.2 years (39 – 79 years)
**Karnofski score**		87 (80 – 90)
**ASA score**		1.8 (1–3)
**BMI**		28.3 kg/m^2^ (20.57 – 41.62 kg/m^2^)
**Hernia type – Giant PEH**	10 (100%)	
Chronic gastric volvulus	5 (50%)	
**HSA**		10.6 cm^2^ (8.1 – 14.4 cm^2^)
**Esophageal lengthening procedure (Collis)**	2 (20.0%)	
**Primary suture of the pillars**		
Primary retroesophageal suture of the pillars	3 (30.0%)	
Plication of the left crus	7 (70.0%)	
**Drainage in the harvesting region**	9 (90.0%)	
No of days		3 days (2 – 4 days)
**Duration of the operation**		203.9 min (160 – 250 min)
**Postoperative complications**		
Mild hematoma on the right thigh	1 (12.5%)	
**Length of postoperative hospital stay**		6.5 days (5 – 8 days)
**30-day death rate**	0	0%
**Follow-up period**		
12 months	5 (50.0%)	
6 months	8 (80.0%)	
**Recurrence rate**	0	0%

The series included 2 (20.0%) males and 8 (80.0%) females. The mean age was 64.2 years (range 39–79 years). The average Karnofsky score was 87 (range 80–90) and ASA score was 1.8 (range 1–3). The average BMI in the selected patients was 28.3 kg/m^2^ (range 20.57–41.62 kg/m^2^). Out of 10 patients with a giant PEH, 5 (50%) presented with chronic gastric volvulus. Due to the secondary short esophagus in 2 (20.0%) patients, the esophageal lengthening procedure had to be performed. The average hiatal surface area (HSA) in our series was 10.6 cm^2^ (range 8.1-14.4 cm^2^). In 3 (30.0%) patients a primary crural repair was performed with retroesophageal cruroraphy using 0 non-absorbable interrupted sutures and in 7 (70.0%) patients the left crus was plicated to normalize crural length, thus permitting a standard hiatal reconstruction. Intraoperative complications included iatrogenic pneumothorax in one patient which was resolved with a chest tube. The average duration of operation was 203.9 min/3.4 hours (range 160 – 250 min).

The subcutaneous drainage was placed in 9 (90.0%) out of 10 patients. The mean duration of the drainage was 3 days (range 2 to 4 days). There was no wound infection. A patient without subcutaneous drainage had a mild hematoma in the harvested region that resolved spontaneously. One patient had exacerbation of the chronic obstructive lung disease. Apart from that, there were no other postoperative complications. The average postoperative length of stay was 6.5 days (5–8 days). Hospital and the 30 days death rate was zero.

Out of 10 patients, 5 completed the annual follow-up visit, while 8 completed 6 months follow-up visit. So far there have been no radiologic and/or symptomatic recurrences and no problems with swallowing function. However, one patient feels a mild discomfort in the harvested region, which however does not influence normal daily activities.

## Discussion

Resolving large hiatal defects still remains a challenge. The primary retroesophageal suture of the diaphragmatic pillars has remained the mainstay of practice for many years [11,15]. Even though there is no consensus, many authors believe that in the cases of large hiatal defects, a structurally altered crura and/or absence of peritoneal lining, a crural reinforcement should be performed [3,4].

Several options have been proposed; amongst them the use of mesh reinforced hiatoplasty. The mesh reinforced hiatoplasty could be performed as an on-lay repair or interposition repair using different types of synthetic mesh i.e. polypropylene, polyester, polytetrafluoroethylen (PTFE) or the combination more suitable for intraperitoneal use [4]. The disadvantages of mesh reinforced hiatoplasty include a possibility of serious complications and also increased costs especially in the usage of PTFE or composite types [16]. The most novel biologic and biosynthetic meshes cause fewer complications [17-19]. However, they adhere less than synthetic ones with a possibility of mesh migration. In addition, they are more expensive than synthetic and have all issues regarding the transfer of heterologous tissue [6].

Recently, a group from Pecs (Hungary) has offered a biologic alternative by performing fascia lata graft hiatoplasty on the animal model [7]. After macroscopic and microscopic evaluation of fascia lata patches following a 6-month follow-up period there were no signs of inflammation, abscess or extensive scar tissue formation. All fascia lata patches were organized and incorporated in scar tissue increasing tissue strength. Also, the shrinkage was up to 20% (only on sides), the neovascularization arriving from diaphragmal blood vessels and peritoneal integration was observed [7].

The first use of the autologous fascia lata graft in the treatment of post-traumatic diaphragmatic hernia was published by Janes in 1931 [8]. In 1968, Brain et al. published the use of the autologous fascia lata graft to create a new phrenoesophageal ligament in the transthoracic repair of hiatal hernia [9]. Autologous fascia lata graft has been used in thoracic surgery for reinforcement of stapled lung resection with excellent results [20].

Encouraged by these results, at our Department we performed 10 laparoscopic autologous fascia lata graft hiatal reinforcements in patients with giant PEHs and a large hiatal defect with the friable crura. The procedure of harvesting autologous fascia lata graft is not technically demanding. It is expected that the main disadvantage of this surgical procedure is cosmetic and includes a scar on the thigh. In addition, the procedure could cause postoperative pain. However, in one case (with no subcutaneous drainage) a mild hematoma occurred but successfully resolved spontaneously. We are not sure whether the discomfort in that specific case was related to the hematoma or procedure itself. No lower limb malfunctioning was observed. It has to be kept in mind that the average age of the patients in our series was 64.2 years. Thus, we could not speculate about the functional result in younger and more active patients. Nevertheless, the average age in this small series does not differ significantly from the average age in the series of 65 giant PEH repairs we performed recently. Indeed, the average age of patients with a giant PEH treated in other series is even higher [11].

Regarding hiatal hernia features, in our series all patients had the giant PEH with at least 1/3 of the stomach positioned intrathoracically [11]. Five patients presented with chronic gastric volvulus. The average HSA in our series was 10.6 cm^2^. After performing primary suture of the pillars by using 0 non-absorbable interrupted sutures we placed 10x8 cm the autologous fascia lata graft in on-lay fashion. The patches of autologous fascia lata should be placed in on-lay fashion after cruroraphy, because the tension strength of the graft, to our opinion, is not sufficient for interposition repair. For the graft fixation we used biodegradable tacks. Based on the animal model, if the initial fixation is good, there is no danger of patch migration after complete scar tissue organization [7].

Except for a mild hematoma that resolved spontaneously, there were no procedure- related complications and the 30 days mortality rate was zero. The average hospital stay of 6.5 days was related to the more detailed monitoring of the patients with new surgical technique, and could be reduced significantly.

Out of 10 patients, 5 completed the annual follow-up visit, while 8 completed 6 months follow-up visit. So far there are no radiologic and symptomatic recurrences (including the problems with swallowing function). However, one patient feels a mild discomfort in the harvested region, which does not influence normal daily activities.

## Conclusion

We present a series of a biologic reinforcement of the large hiatal defects with the friable crura in the patients with a giant PEH by using an autologous fascia lata graft. For the present follow-up period, no hiatal hernia recurrence or procedure-related significant complications have been observed. Autologous fascia lata graft hiatal reinforcement represents a technically feasible, easy, and available option for the on-lay reinforcement of large hiatal defects with the friable crura in the laparoscopic repair of giant PEHs. However, due to a relatively small number of patients and short follow-up period we express the need for further evidence regarding this surgical technique.

### IRB approval of the protocol

This technique and study was approved by the IRB of the Clinical Center of Serbia.

## Abreviations

PEH: Paraesophageal hernia
PTFE: Polytetrafluoroethylen
HSA: Hiatal surface area
